# Ustekinumab versus vedolizumab in patients with Crohn’s disease refractory to anti-tumour necrosis factor: A systematic review and meta-analysis

**DOI:** 10.12669/pjms.41.7.12141

**Published:** 2025-07

**Authors:** Jianfeng Dai, Rui Guo, Jing Gong

**Affiliations:** 1 Jianfeng Dai, College of Agroforestry and Health, The Open University of Sichuan, Chengdu, Sichuan Province 610073, P.R. China; 2 Rui Guo, Dept. of Gastroenterology, Sichuan Provincial People’s Hospital, School of Medicine, University of Electronic Science & Technology of China, Chengdu, Sichuan Province 610072, P.R. China; 3 Jing Gong, College of Agroforestry and Health, The Open University of Sichuan, Chengdu, Sichuan Province 610073, P.R. China

**Keywords:** Anti-TNF resistance, Biologics, Crohn’s disease, Meta-analysis, Second line therapy, Systematic review, Ustekinumab, Vedolizumab

## Abstract

**Objective:**

To compare clinical efficiency of Ustekinumab (UST) and Vedolizumab (VDZ) in patients with Crohn’s disease (CD), refractory to anti-tumour necrosis factor (anti-TNF) therapy.

**Methods:**

PubMed, Web of Science, Scopus, and Embase databases were searched for studies published from inception until 15th May 2024. Cohort studies comparing UST and VDZ regimens in patients with refractory CD and reporting clinical, steroid-free, and biological remission, as well as providing data on treatment persistence were included. Random-effects models were used, and the meta-analyses results were presented as odds ratios (ORs) with 95% confidence intervals (CIs).

**Results:**

Sixteen included studies with 6584 patients were analysed. UST treatment regimen was linked to significantly higher clinical remission rates at 14-16 weeks (OR 1.41, 95% CI: 1.01, 1.98) but not at 52 weeks (OR 1.24, 95% CI: 0.85, 1.81) compared to VDZ. Patients receiving UST had higher steroid-free remission (SFR) rates in both induction (OR 1.33, 95% CI: 1.02, 1.73) and maintenance phases of the treatment (OR 1.56, 95% CI: 1.16, 2.08). However, biological remission rates during both induction and maintenance phases were comparable in the two groups. UST was associated with lower risk of all-cause hospitalization (OR 0.72, 95% CI: 0.59, 0.88) compared to VDZ.

**Conclusion:**

UST is more efficient than VDZ in achieving rapid clinical remission and sustained steroid-free remission in CD patients who are refractory to anti-TNF therapy. While both regimens achieve long-term control of the disease with similar safety profiles, UST resulted in a lower risk of hospitalization. Further studies should confirm long-term outcomes and cost-effectiveness of these treatment plans.

## INTRODUCTION

Crohn’s disease (CD) is a chronic inflammatory bowel disease.^1^ Despite advances in the management of CD, a significant proportion of patients do not achieve acceptable disease control with conventional therapies, such as corticosteroids, immunomodulators, and anti-tumour necrosis factor (TNF) agents.^2^-^4^ Anti-TNF agents, such as infliximab and adalimumab, have become a cornerstone of CD treatment, and have demonstrated significant efficacy in both inducing the remission and its maintenance in patients with moderate to severe CD.^5^,^6^

However, despite its effectiveness, approximately 10% to 40% of patients exhibit primary non-response to anti-TNF therapy. Moreover, patients who initially respond to the regimen are still at risk of loss of response.^7^ Anti-TNF treatment is also associated with adverse events that contribute to treatment discontinuation.^8^,^9^ Ustekinumab (UST) and vedolizumab (VDZ) have emerged as promising biologic therapies for CD patients with refractory disease.^10^ UST is a monoclonal antibody targeting the p40 subunit of interleukin-12 (IL-12) and interleukin-23 (IL-23) that are implicated in the pathogenesis of CD. VDZ is a monoclonal antibody that targets the α4β7 integrin, thereby inhibiting the migration of gut-homing T lymphocytes to the inflamed intestinal tissue, a mechanism highly specific to the gut.^11^

The efficiency of both UST and VDZ in both anti-TNF-naive and experienced CD patients have been validated through randomized placebo-controlled trials.^12^-^17^ However, no head-to-head clinical trials directly comparing UST and VDZ have been conducted to date. Network meta-analyses of randomized trials have been largely inconclusive, underscoring the need for more direct comparative research.^18^,^19^ Recently, several observational studies have attempted to address this gap by comparing the outcomes of UST and VDZ in CD patients.^20^-^23^ This study intended to evaluate the comparative efficacy and safety of UST and VDZ in CD patients who are refractory to anti-TNF therapy. The primary outcome of interest was clinical remission. Secondary outcomes included steroid-free remission (SFR), biological remission, and treatment persistence.

## METHODS

This systematic review and meta-analysis was conducted in accordance with the PRISMA 2020 guidelines. 24 The study protocol was preregistered in the PROSPERO database (CRD42024547265).

### Inclusion criteria:

- Studies of adult (18 years and older) patients, diagnosed with moderate to severe CD.
- Studies with patients who have previously been treated with anti-TNF agents and were either primary non-responders or stopped response to these therapies after initial response.
- Studies, specifically comparing efficacy of UST and VDZ.
- Randomized controlled trials (RCTs) and observational (cohort and case-control) studies.
- Studies that reported on at least one relevant clinical outcome such as clinical remission, steroid-free remission (SFR), biological remission and treatment persistence.
- English-language studies with full-text availability.
- Studies with both adjusted and unadjusted analyses. This approach allows to perform primary analyses based on studies that had reported confounder adjusted estimates or had done propensity score matching or used Inverse probability of treatment weighting (IPTW) approach to balance baseline patient characteristics.^25^

### Exclusion criteria:

- Studies that lacked clear documentation of prior anti-TNF treatment failure.
- Studies that did not provide disaggregated data for UST and VDZ when both were included in broader comparisons.
- Studies with insufficient data to extract relevant outcomes or conduct meaningful analysis
- Reviews, editorials, conference abstracts and case report.
- Studies that focussed solely on pharmacokinetics, pharmacodynamics, or molecular mechanisms without clinical outcome data.

### Information sources and search strategy:

PubMed, Web of Science, Scopus, and Embase databases were searched for studies published from inception of these databases until 15th May 2024. The search strategy was developed using a combination of MeSH terms and free-text keywords and was customized for each database to account for differences in indexing systems and available search filters. While the core concepts remained consistent, database-specific adaptations were made to ensure comprehensive retrieval of relevant studies. Search keywords used were: (“Crohn’s disease”[MeSH Terms] OR “Crohn disease” OR “regional enteritis”) AND (“Ustekinumab”[MeSH Terms] OR “Stelara” OR “IL-12/23 inhibitor”) AND (“Vedolizumab”[MeSH Terms] OR “Entyvio” OR “integrin antagonist”) AND (“anti-TNF refractory” OR “TNF-alpha inhibitor failure” OR “TNF therapy resistant”) AND (“clinical remission” OR “steroid-free remission” OR “corticosteroid-free remission” OR “biological remission” OR “mucosal healing” OR “endoscopic remission” OR “treatment persistence” OR “drug adherence” OR “treatment continuation”).

### Selection process:

The study selection process involved two levels of screening: primary (title and abstract) screening and secondary (full-text) screening. After deduplication of records identified through the database searches, two reviewers (JD and JG) independently screened the titles and abstracts of the remaining studies to identify potentially relevant articles (primary screening). Studies deemed relevant were then retrieved in full text and independently assessed by the same reviewers (JD and JG) for eligibility based on the predefined inclusion and exclusion criteria (secondary screening). Any discrepancies in article screening and selection were resolved through discussion between the two review authors, with the senior author (RG) facilitating the process to reach a mutual consensus.

### Data collection process:

Two authors (JD and JG) independently used a structured extraction form to collect information such as study identifiers (author and publication year), study design, location, age and sex of the study participants, duration of follow up, use of steroids and immunosuppressants and sample size along with important findings. It is worthwhile to mention that no attempts were made to contact study authors for missing data. The majority of the information required for data extraction was available in the published articles or their supplementary materials. Only data from these sources were used to maintain transparency and ensure the replicability of the review process. Any discrepancies or disagreements were resolved through discussion, facilitated by the senior author (RG).

### Risk of bias assessment:

Quality of studies was assessed using the Newcastle-Ottawa Scale (NOS), with a maximum score of nine points.^26^ This scale evaluates the risk of bias across three domains: selection of study groups (maximum four points), comparability of groups (maximum two points), and assessment of outcome or exposure (maximum three points). Each study was independently reviewed and scored by two authors (JD and JG), with any discrepancies resolved through discussion, facilitated by the senior author (RG). The total NOS score ranges from 0 to 9, with higher scores indicating better quality. Studies scoring between seven and nine were considered to have a low risk of bias, those scoring 4 to 6 were classified as having a moderate risk, and studies scoring below four were considered to have a high risk of bias.

### Outcomes:

The primary outcome of interest was clinical remission. Secondary outcomes included steroid-free remission (SFR), biological remission, and treatment persistence.

### Statistical analysis:

Pooled effect size was reported as odds ratios (OR) with 95% confidence intervals (CIs). Analysis was done using a random-effects model.^27^ The analysis included studies that had either reported confounder-adjusted estimates or had done propensity score matching or used inverse probability of treatment weighting (IPTW) approach to balance baseline patient characteristics. Statistical heterogeneity across studies was assessed using the I² statistic. I² value of 50% or more was considered to represent moderate to high heterogeneity.^27^ In cases where moderate to high heterogeneity (I² > 50%) was observed, the results were interpreted with appropriate caution. Publication bias was assessed by Egger’s test.^28^ P<0.05 was considered statistically significant. All statistical analyses were conducted using STATA version 15.0.

## RESULTS

**Fig.1:**
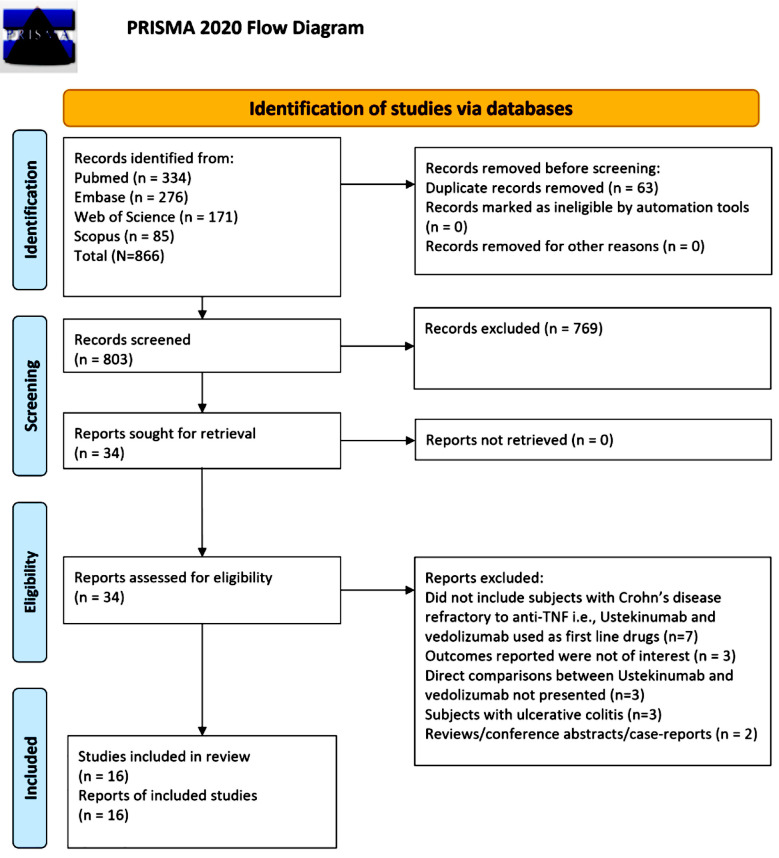
PRISMA flowchart to show process of study selection.

The database search identified 866 relevant studies. Ultimately, 16 studies were included in the final meta-analysis (Supplementary Table-I, Fig.1).^20^-^23^,^29^-^40^ Most of the included studies (n=14) had retrospective cohort design. Studies were conducted across different geographical locations (Supplementary Table-I). Mean age of the participants in the included studies ranged from 35 to 50 years. Almost all studies had a follow up period of at least 12 months. Most (n=12) had reported confounder-adjusted effect sizes or had done propensity score matching at baseline or used IPTW approach to balance baseline patient characteristics. One study by Ibing et al (2023) reported separate findings from two different cohorts in one paper.^31^ Therefore, in the analysis, findings of each of these cohorts was used separately and labelled as Ibing et al (2023) (A) and Ibing et al (2023) (B). The included studies contributed to a total sample of 6584 CD patients with 4013 in the UST group and 2571 in the VDZ group. All included studies were of good quality i.e., with low risk of bias, as indicated by the NOS score. There were nine studies with a score of 8 and seven studies with a score of seven.

**Supplementary Table-I:** Included studies with important characteristics.

Author	Study design; location	Population	Follow up duration	Use of steroids at baseline	Use of immune-suppressant at baseline	Sample size	Quality score
Shimazaki et al (2024)	RC; Japan	Mean age 39 years Male (75%)	At least 12 months	UST: 28.2% VDZ: 33.3%	UST: 23.2% VDZ: 27.8%	UST (n=220) VDZ (n=36)	7
García et al (2024)	RC; Spain	Mean age 46 years Male (50%)	Median 4.7 years for VDZ; 2.8 years for UST	UST: 22% VDZ: 37%	UST: 31% VDZ: 37%	UST (n=628) VDZ (n=207)	8
Kapizioni et al (2024)	RC; United Kingdom	Mean age 36 years Female (56%)	Over 3 years	UST: 3.8% VDZ: 3.5%	---	UST (n=425) VDZ (n=743)	7
Kappelman et al (2023)	RC; USA	Mean age 43 years Female (56%)	455 days	UST: 27.3% VDZ: 26.2%	UST: 43.4% VDZ: 42.5%	UST (n=884) VDZ (n=484)	8
Ibing et al (2023)	RC; USA	MSHS cohort Mean age 42 years Male (44%) SPARC cohort Mean age 41 years Proportion male lower in UST group (39% vs. 53%)	MSHS cohort Median follow up 3.2 years SPARC cohort Median follow up 3.8 years	MSHS cohort UST: 53.8% VDZ: 59.5% SPARC cohort UST: 59.0% VDZ: 66.7%	MSHS cohort UST: 43.4% VDZ: 40.5% SPARC cohort UST: 48.2% VDZ: 41.7%	MSHS cohort UST (n=145) VDZ (n=74) SPARC cohort UST (n=139) VDZ (n=72)	8
Yang et al (2023)	RC; China	Mean age 34 years Higher proportion of male in UST group (75 vs. 63%)	52 weeks	UST: 6% VDZ: 6.7%	UST: 1.8% VDZ: 1.3%	UST (n=386) VDZ (n=150)	8
Alrashed et al (2023)	RC; Kuwait	Mean age 35 years Male (52%)	52 weeks	---	Overall: 31%	UST (n=101) VDZ (n=29)	7
Onali et al (2022)	RC; Italy	Mean age lower in UST group (41 vs. 47 years) Proportion male higher in UST group (54% vs. 26%)	12 months	UST: 31.4% VDZ: 49.4%	UST: 9.6% VDZ: 9.5%	UST (n=239) VDZ (n=231)	8
Rayer et al (2022)	RC; France	Mean age of 40 years Male (44%)	Mean 118 weeks	UST: 32% VDZ: 22%	UST: 19% VDZ: 15%	UST (n=90) VDZ (n=42)	8
Hyun et al (2022)	RC; Republic of Korea	Mean age of 36 years Proportion male lower in UST group (44% vs. 54%)	48 weeks	UST: 43.8% VDZ: 71.4%	UST: 87.5% VDZ: 57.1%	UST (n=16) VDZ (n=28)	7
Bacsur et al (2022)	RC; Hungary	Median age 36 years Male (35%)	52 weeks	UST: 27.9% VDZ: 38.5%	UST: 21.1% VDZ: 29.2%	UST (n=161) VDZ (n=65)	8
Manlay et al (2021)	RC; France	Mean age lower in UST group (37 vs. 41 years) Proportion female (62%)	16.5 months	UST: 26.3% VDZ: 31.8%	UST: 14.3% VDZ: 19.3%	UST (n=224) VDZ (n=88)	7
Townsend et al (2020)	PC; UK	Mean age lower in UST group (42 vs. 44 years) Proportion male lower in UST group (31% vs. 44%)	12 months	UST: 44.4% VDZ: 35.3%	UST: 55.6% VDZ: 49.4%	UST (n=45) VDZ (n=85)	7
Alric et al (2020)	RC; France	Mean age of 40 years Male (46%)	48 weeks	UST: 28% VDZ: 48.5%	UST: 23.4% VDZ: 42.4%	UST (n=132) VDZ (n=107)	8
Biemans et al (2020)	PC; Netherlands	Median age of 37 years Male (37%)	Median 104 weeks	UST: 11.8% VDZ: 31.3%	UST: 23.5% VDZ: 18.8%	UST (n=128) VDZ (n=85)	8
Kolar et al (2019)	RC; Czech Republic	Mean age of 40 years Higher proportion of male in UST group (48 vs. 25%)	32 weeks	UST: 32% VDZ: 20%	UST: 44% VDZ: 46.7%	UST (n=50) VDZ (n=45)	7

UST: Ustekinumab; VDZ: Vedolizumab; RC: retrospective cohort; PC: prospective cohort; UK: United Kingdom; MSHS: Mount Sinai Health System (MSHS); SPARC: Study of a Prospective Adult Research Cohort.

### Clinical remission:

**Fig.2:**
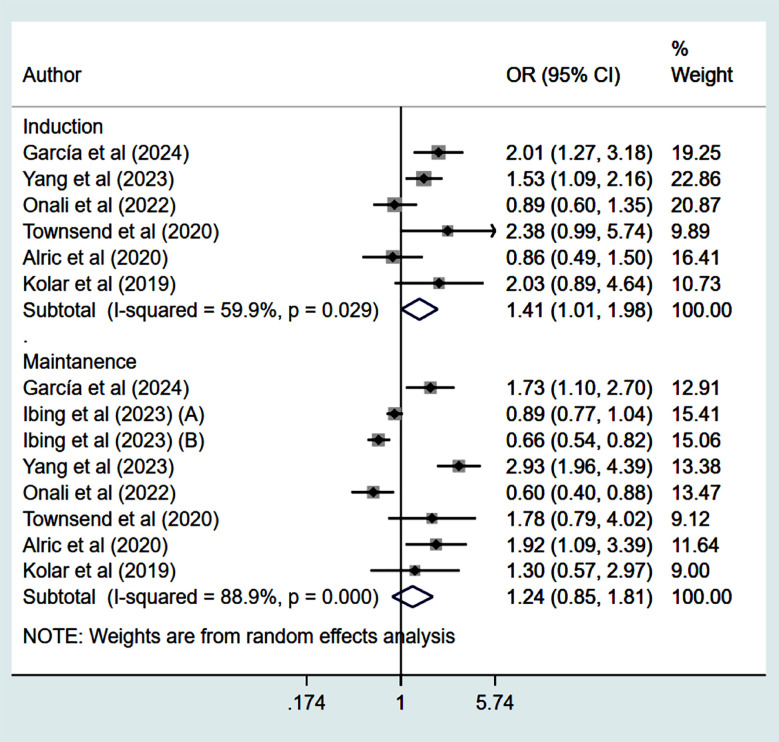
Clinical remission among those on Ustekinumab, compared to those on Vedolizumab.

**Supplementary Fig.1:**
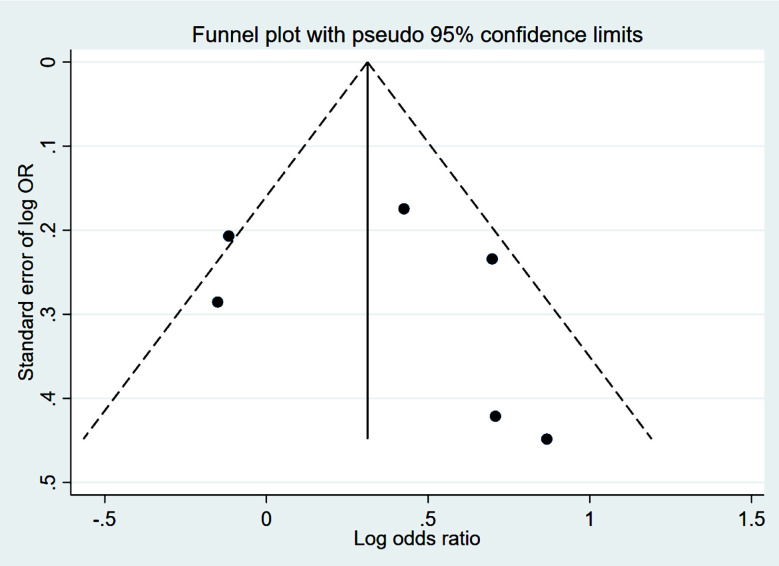
Funnel plot for publication bias related to comparison of clinical remission, between Ustekinumab and vedolizumab, during indction phase in subjects with Crohn’s disease, refractory to anti-tumour necrosis factor

**Supplementary Fig.2:**
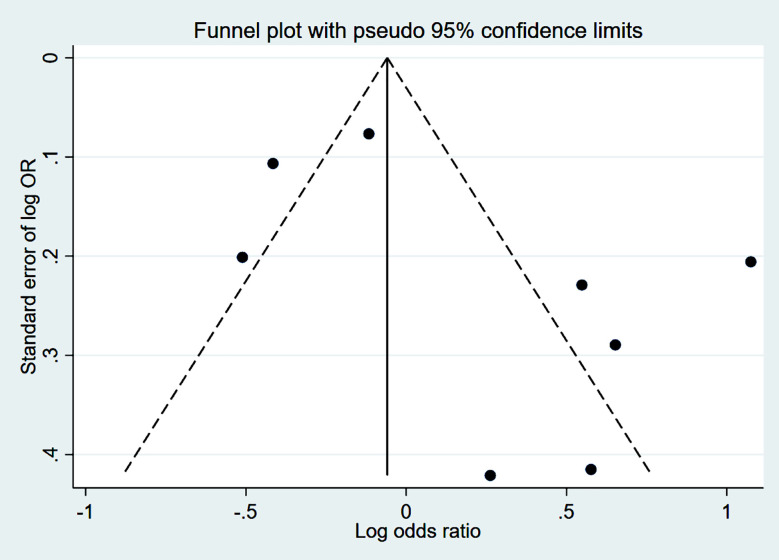
Funnel plot for publication bias related to comparison of clinical remission, between Ustekinumab and vedolizumab, during maintenance phase in subjects with Crohn’s disease, refractory to anti-tumour necrosis factor.

Studies that had propensity score matching and/or adjusted effect sizes were pooled. Compared to patients who received VDZ, patients who were treated with UST had higher rates of clinical remission (CR) at 14-16 weeks (induction phase) (OR 1.41, 95% CI: 1.01, 1.98; n=6, I^2^=59.9%) (Fig.2). However, the remission rate was similar in the two groups at 52 weeks (maintenance phase) (OR 1.24, 95% CI: 0.85, 1.81; n=8, I^2^=88.9%) (Fig.2), with no apparent publication bias on Egger’s test (p=0.59 at 14-16 weeks and p=0.16 at 52 weeks) and on visual inspection of funnel plots (Supplementary figures 1 and 2).

### Steroid-free remission:

**Fig.3:**
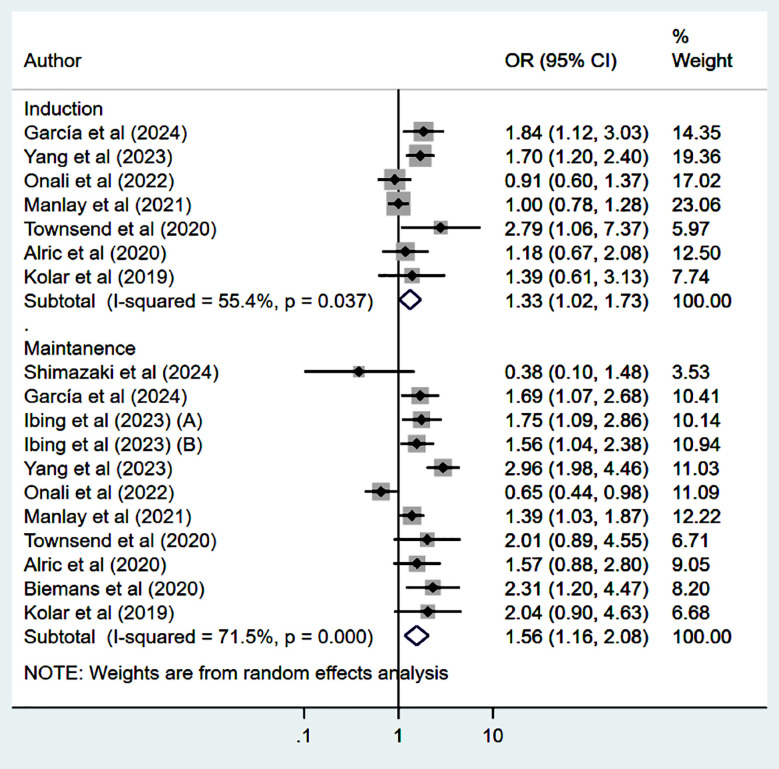
Steroid free remission among those on Ustekinumab, compared to those on Vedolizumab.

Patients treated with UST had higher and statistically significant steroid-free remission (SFR) at 14-16 weeks (induction phase) (OR 1.33, 95% CI: 1.02, 1.73; n=7, I^2^=55.4%) and at 52 weeks (maintenance phase) (OR 1.56, 95% CI: 1.16, 2.08; n=11, I^2^=71.5%) (Fig.3) compared to the VDZ group. There was no evidence of publication bias on Egger’s test (p=0.22 at 14-16 weeks and p=0.93 at 52 weeks).

### Biological remission:

**Fig.4:**
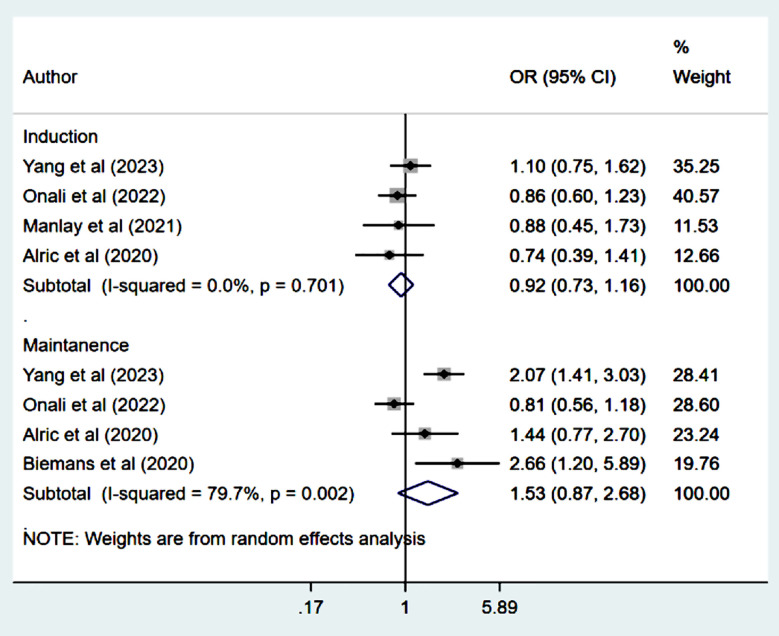
Biological remission among those on Ustekinumab, compared to those on Vedolizumab.

Odds of biological remission during the induction phase (14-16 weeks) (OR 0.92, 95% CI: 0.73, 1.16; n=4, I^2^=0.0%) as well as the maintenance phase (52 weeks) (OR 1.53, 95% CI: 0.87, 2.68; n=4, I^2^=79.7%) were comparable in both groups (Fig.4). There was no evidence of publication bias on Egger’s test (p=0.53 at 14-16 weeks and p=0.58 at 52 weeks).

### Treatment persistence:

**Fig.5:**
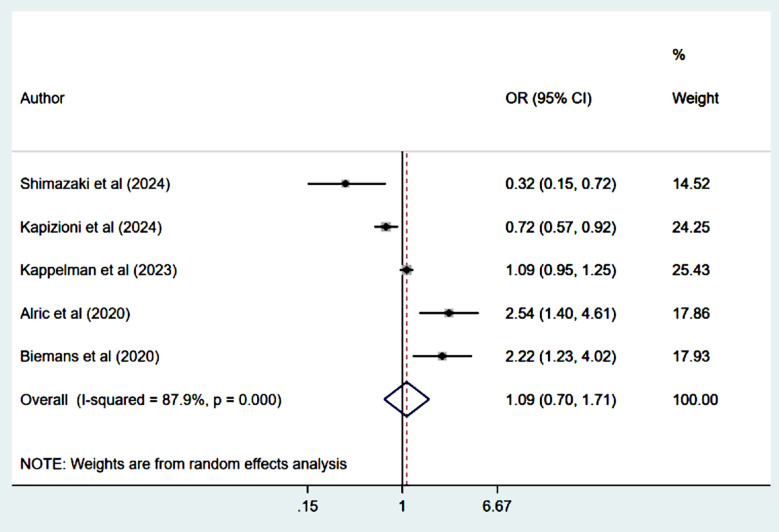
Treatment persistence among those on Ustekinumab, compared to those on Vedolizumab.

Persistence of treatment rate which was measured in all studies at 52 weeks was comparable in all groups of patients (OR 1.09, 95% CI: 0.70, 1.71; n=5, I^2^=87.9%) (Fig.5). There was no evidence of publication bias on Egger’s test (p=0.89).

### Adverse events, including hospitalization:

Incidence of adverse events (OR 0.64, 95% CI: 0.33, 1.23; n=4, I^2^=73.9%) was statistically comparable between the two groups. The commonly reported adverse events were joint pain (arthralgia), recurrent infections, skin disorders including genital warts, oral herpes and headache. UST regimen was linked to a lower risk of all-cause hospitalization (OR 0.72, 95% CI: 0.59, 0.88; n=3, I^2^=0.0%), compared to VDZ regimen. There was no evidence of publication bias on Egger’s test (p=0.10 for adverse events and p=0.78 for all-cause hospitalization).

## DISCUSSION

In this meta-analysis, we included 16 studies comprising 6,584 patients to compare the efficacy and safety outcomes of UST and VDZ treatment regimens. Our findings indicate that UST was associated with significantly higher clinical remission rates at 14-16 weeks, compared to VDZ; however, this difference was not observed at 52 weeks. Patients receiving UST demonstrated higher steroid-free remission (SFR) rates in both the induction and maintenance phases. In contrast, biological remission rates remained comparable between the two treatment groups throughout both phases. Additionally, UST was associated with a lower risk of all-cause hospitalization compared to VDZ, suggesting a potential advantage in reducing healthcare utilization.

### Comparison with previous meta-analysis:

A review by Parrot et al. analysed five studies involving 1026 patients,^41^ and showed that at week 14, both UST and VDZ regimens were associated with similar rates of clinical remission, SFR, and biological remission. However, these rates were significantly higher in patients who received UST at week 52. Similar findings were reported by the systematic review by Sharip et al.^42^ While our study also demonstrated that UST regimen is associated with higher SFR during the maintenance phase, there are notable differences between our results and the previous reports. Our study included a larger number of contemporary studies with a higher sample size, capturing more heterogeneity in patient populations, disease severity, and treatment protocols. Additionally, variations in follow-up durations, differences in induction and maintenance dosing strategies, and evolving clinical practices may have influenced the observed outcomes. Furthermore, inconsistent definitions of clinical remission, SFR, and biological remission across studies could have contributed to variations in reported effectiveness.

### Possible explanations for the observed findings:

UST is a monoclonal antibody that targets the p40 subunit of interleukins 12 and 23 (IL-12 and IL-23) that are involved in the inflammatory pathways of CD.^11^,^43^ This mechanism may explain the rapid induction of clinical remission and SFR observed in our analysis. The ongoing blockade of IL-12 and IL-23 pathways also supports the sustained benefits seen during the maintenance phase. VDZ is an integrin receptor antagonist that selectively binds to the α4β7 integrin, inhibiting the interaction between α4β7 and mucosal addressin cell adhesion molecule-1 (MAdCAM-1).^11^,^44^,^45^ This mechanism prevents memory T-lymphocytes migration into gastrointestinal tissues, thereby reducing gut-specific inflammation. While VDZ is effective in inducing and maintaining the remission in CD, its slower onset of action compared to UST may account for the differences observed in the induction phase. However, its gut-selective mechanism supports long-term efficacy, which aligns with the comparable clinical remission rates seen at 52 weeks.

The superior SFR in patients who were treated with UST during both induction and maintenance phases can be attributed to the broad and rapid anti-inflammatory effect of UST through the inhibition of IL-12 and IL-23.^11^,^43^ This mechanism provides more comprehensive control of both systemic and local inflammation, reducing the need for corticosteroids to manage disease activity.^46^ In contrast, VDZ’s more targeted approach to gut-specific inflammation may result in slower and less comprehensive inflammatory control, leading to a greater reliance on steroids to achieve and maintain remission.^47^,^48^

Similar biological remission rates of both regimens can be explained by their effective targeting of different but complementary pathways in the inflammatory process of CD. Lower risk of all-cause hospitalization observed in patients receiving UST, compared to VDZ can be attributed to the rapid and comprehensive anti-inflammatory effect of UST, sustained long-term control of inflammation, superior steroid-sparing benefits, and systemic impact on both intestinal and extra-intestinal manifestations of CD.

### Importance of the findings to the existing medical literature:

Our study demonstrates that UST offers significant advantages in terms of steroid-free remission during both induction and maintenance phases, along with a lower risk of all-cause hospitalization. These findings are particularly relevant for clinicians managing patients with CD who have failed anti-TNF therapy. The observed differences in remission rates and hospitalization risk highlight the potential benefits of UST for achieving faster and more sustained disease control.

### Strengths:

A major strength of our meta-analysis is the inclusion of a larger number of contemporary studies, allowing for a more robust comparison between UST and VDZ. We accounted for variations in study design and patient characteristics by including only studies with appropriate confounder adjustments or propensity score matching, reducing the risk of bias. Furthermore, our study provides a more detailed examination of different outcome measures, including clinical, steroid-free, and biological remission rates, as well as hospitalization risk-offering a comprehensive evaluation of both efficacy and safety.

### Limitations:

First, the included studies exhibit considerable heterogeneity in terms of patient populations, and definitions adopted for reporting of the outcome measures. This variability may affect the generalizability of our findings. While we tried to address some of the issue by including studies with confounder adjustments or propensity score matching, the possibility of unmeasured confounders remains, which could bias the overall findings. Second, our meta-analysis primarily relies on comparisons between UST and VDZ using secondary data, as head-to-head randomized controlled trials (RCTs) are lacking. Third, most studies had relatively short follow-up durations, typically up to 52 weeks. Fourth, there is a lack of standardization in the definitions of clinical remission, SFR, and other outcome measures across studies. This variability can complicate the interpretation and comparison of the results.

### Implications of the study findings:

The findings of this meta-analysis have important clinical implications for the management of Crohn’s disease in patients refractory to anti-TNF therapy. The superior performance of Ustekinumab in achieving early clinical remission and sustained steroid-free remission suggests it may be a more effective option for inducing and maintaining disease control in this difficult-to-treat population. The observed lower risk of all-cause hospitalization with UST further supports its potential benefit in reducing healthcare utilization and improving patient outcomes. Given that biological remission rates and long-term disease control were comparable between the two agents, treatment decisions may also need to consider factors such as patient preference, administration routes, and cost. These results underscore the need for individualized treatment strategies and highlight the importance of future research to evaluate long-term efficacy, safety, and cost-effectiveness of UST versus VDZ in real-world settings.

## CONCLUSION

Our meta-analysis showed that UST may offer advantages over VDZ in terms of achieving rapid clinical remission and sustained SFR in CD patient’s refractory to anti-TNF therapy. Both treatments were effective in maintaining long-term disease control and had similar safety profiles. However, UST regimen was linked to lower risk of all-cause hospitalization and may provide additional clinical benefits. Future studies should further investigate the long-term outcomes and cost-effectiveness of these therapies to support comprehensive care for CD patients.

### Future research directions:

First, head-to-head randomized controlled trials are needed to validate our findings. Second, long-term follow-up studies are necessary to assess the durability of treatment effects beyond 52 weeks, particularly in terms of disease progression, sustained remission, and cost-effectiveness. Additionally, future research should focus on refining treatment algorithms by identifying patient subgroups that may derive greater benefit from one therapy over the other based on biomarkers, disease severity, or prior treatment history.

### Authors’ contributions:

**JD:** Study design, literature search and manuscript writing. Revision and validation

**JD, RG and JG:** Data collection, data analysis, interpretation and Critical Review.

All authors have read and approved the final manuscript. They are all responsible for the integrity of the study.
